# Implication of COVID-19 on Erythrocytes Functionality: Red Blood Cell Biochemical Implications and Morpho-Functional Aspects

**DOI:** 10.3390/ijms23042171

**Published:** 2022-02-16

**Authors:** Annamaria Russo, Ester Tellone, Davide Barreca, Silvana Ficarra, Giuseppina Laganà

**Affiliations:** 1 Istituto Comprensivo Primo, 98057 Milazzo, Italy; annamaria.russo81@posta.istruzione.it; 2 Department of Chemical, Biological, Pharmaceutical and Environmental Sciences, University of Messina, Viale Ferdinando Stagno d’Alcontres 31, 98166 Messina, Italy; silvana.ficarra@unime.it (S.F.); giuseppina.lagana@unime.it (G.L.)

**Keywords:** SARS-CoV-2, red blood cells, COVID-19, hemoglobin, infections, hematology

## Abstract

Several diseases (such as diabetes, cancer, and neurodegenerative disorders) affect the morpho-functional aspects of red blood cells, sometimes altering their normal metabolism. In this review, the hematological changes are evaluated, with particular focus on the morphology and metabolic aspects of erythrocytes. Changes in the functionality of such cells may, in fact, help provide important information about disease severity and progression. The viral infection causes significant damage to the blood cells that are altered in size, rigidity, and distribution width. Lower levels of hemoglobin and anemia have been reported in several studies, and an alteration in the concentration of antioxidant enzymes has been shown to promote a dangerous state of oxidative stress in red blood cells. Patients with severe COVID-19 showed an increase in hematological changes, indicating a progressive worsening as COVID-19 severity progressed. Therefore, monitored hematological alterations in patients with COVID-19 may play an important role in the management of the disease and prevent the risk of a severe course of the disease. Finally, monitored changes in erythrocytes and blood, in general, may be one of the causes of the condition known as Long COVID.

## 1. Introduction

The exchange of gases between the tissues and lungs and the maintenance of the acid-base balance represents the main goal played by the circulatory system. Red blood cells (RBCs) are the major cellular components of the blood, and, for this reason, their role is essential not only to ensure the right supply of oxygen to the tissues and the concomitant excretion of carbonic dioxide (CO_2_) but also to define the biophysical consistency of the blood and the efficiency of the entire bloodstream. To best perform their oxygen transport function, mature RBCs are deprived of the nucleus and organelles to make way for hemoglobin (Hb), the main protein responsible for oxygen transport. Hb is a tetrameric molecule floating between two conformational states, T at low oxygen affinity and R high oxygen affinity [1]. The oxygen binding and release by Hb are strictly modulated by a complex of homo- and heterotrophic factors, including the pH of blood; at low oxygen pressure, the CO_2_ dissociation promotes blood acidification with consequent T state stabilization and oxygen release from Hb. The oxygen release is also effectively facilitated by CO_2_ and 2,3-bisphosphoglyceric acid, both contributing directly to the main and most studied activity of RBCs: the oxygen transport from the lungs to tissues and carbon dioxide from the tissues to the lungs, where it will be exhaled. 

In addition to their canonical role as gases and nutrients transporters, being cells in close contact with oxygen, RBCs possess a very efficient antioxidant system that preserves their integrity. They are also important modulators of nitric oxide (NO) metabolism and participate in the control of blood rheology via their concentration (hematocrit), which critically and specifically defines the viscosity of the blood.

Pathological alterations of RBCs disturbing their cellular function and/or deformability have been associated with several diseases, such as diabetes, sickle cell anemia, malaria, and some neurodegenerative diseases [2,3,4,5,6,7,8]. In this article, we will specifically evaluate some functional and structural alterations of RBCs related to COVID-19 disease. In fact, despite the development of vaccines, the worldwide data show that the pandemic is not yet under control and further studies need to better understand the biology of viral infection. In addition, the persistent post-infection symptomatology reported by several healed COVID-19 patients requires a more accurate and extensive study focused on the hematological changes related to SARS-CoV-2 infection. The work carried out in this review aims to highlight the combination of hematological and erythrocyte dysregulation mechanisms that favor the evolution of SARS-CoV-2 infection in order to inspire the scientific community and enhance therapeutic approaches against COVID-19.

### 1.1. Coronavirus Disease 

COVID-19 is an infectious disease. Its acronym COronaVIrus Disease (19 indicates the year during which the virus was first identified) derives from its viral origin. In fact, COVID-19 is caused by a virus called SARS-CoV-2, belonging to the Coronaviridae family, a group of enveloped positive-sense single-stranded RNA viruses that cause gastrointestinal and respiratory infections [9,10]. The new Coronavirus, responsible for the current pandemic, emerged in Wuhan, China, and is also formally called SARS-CoV-2 (Severe Acute Respiratory Syndrome Coronavirus 2) to distinguish it from SARS-CoV and MERS-CoV (Middle East Respiratory Syndrome Coronavirus) [11]. Coronaviruses, in general, are known to infect the bronchial epithelium cells and the superficial respiratory tract; they can be the cause of severe pathologies and serious lung damage, leading, in some cases, to death from air deficiency. The new Coronavirus, responsible for COVID-19, causes the most acute respiratory pathology with multiorgan complications and distinctive symptoms, such as cough, fever, headache, astheny, difficulty breathing, and gastrointestinal disorders [12]. In addition, peculiar transient COVID-19 symptoms are anosmia (lack of smell) and ageusia (lack of taste). 

### 1.2. Coronavirus

The Coronavirus name derives from the characteristic crown shape conferred to the virus by the proteins on its outer shell, known as spikes (thorns). Spike proteins favor the virus infection to human cells as they have a sort of molecular hook (RBD = Receptor Binding Domain) with which the viral spike proteins bind to Angiotensin-converting enzyme 2 (ACE2) receptors [13]. This last is an important regulator of blood pressure, and it is expressed in the lung and on the epithelial cells of the small intestine. The ACE2 expression in the small intestine could explain the potential fecal–oral transmission of the virus, in addition to respiratory transmission. RBD-ACE2 binding is essential to initiate infection but, the virus also exploits the sialic acid receptor present in the tissues of the upper respiratory tract. From a structural point of view, the corona of the virus consists of four proteins; from the outside: spike, envelope, membrane, and nucleocapsid, this last surrounding the RNA [14]. The viral complex incorporation takes place by the conformational change of the spike protein induced by the binding between RBD and the ACE2 receptor of the host cell: the conformational change of the spike protein approaches the viral membrane to the plasma membrane of the host cell, up to the interaction, the fusion between membranes and, finally, the incorporation of the infecting virus. Once the viral genome is inside the host cell, the virus begins its replication, and the infection process can be considered complete. SARS-CoV-2 “appropriates” the host’s ribosomes and exploits them to translate its genome and synthesizes proteins necessary for viral replication and assembly of new virions. With the transcription and replication of the viral genome, SARS-CoV-2 begins to spread in the host, starting the real infectious disease [15].

### 1.3. Clinical Aspects of COVID-19 Infection

The infection can start asymptomatic, or it manifests with common influenza symptoms (fever, cough), gastrointestinal disorders, loss of taste and smell, or conjunctivitis. Some COVID-19 patients may develop dyspnea and severe respiratory failure due to the development of interstitial pneumonia with high fever, myocardial damage, and risk of death [15]. The respiratory failure complication derives from the virus’ entry into the pulmonary alveoli; the human immune system counteracts this invasion, activating a strong inflammatory response with rapid and important release of interleukin-6 and other pro-inflammatory cytokines, such as chemokines, tumor necrosis factor-α, and interleukina-1β [16]. The picture of “cytokine storm” is configured, which can cause a serious insufficiency of several organs and lead to the death of the patient [17]. Therefore, “acute respiratory distress syndrome” characterized by diffuse alveolar damage, shock, and acute renal failure is the most frequent complication of viral infection. In addition, some COVID-19 patients experience alterations in blood clotting resulting in severe thromboembolic events [18].

## 2. COVID-19 and RBCs Metabolism

Many Coronavirus patients have acute lung involvement, accompanied by hypoxia and dyspnea as alarming symptoms. Others experience a seemingly well state, in which the lungs do not appear severely damaged despite insufficient blood oxygenation (silent hypoxia) [19]. These conditions have led several research groups to suppose a direct involvement of erythrocytes in Coronavirus infections [20,21,22]. Cosic et al., in 2020, through studies performed with the Resonant Recognition Model (RRM), proposed RBCs as an alternative point of access to the SARS-CoV-2 virus, which, by entering through the alveoli membrane in the lung, reaches the bloodstream [21]. In the blood, SARS-CoV-2 infects RBCs through the binding between S1 Spike protein and Band-3 protein on the erythrocyte membrane [20]. Moreover, Band-3 has already been indicated as a binding receptor for protozoan parasites, such as Plasmodium falciparum [23]. The link between Band-3 and the virus does not support viral replication but can affect different characteristics of the RBC, also linked to its functionality (as well as the release of oxygen). In this context, studies on RBCs from COVID-19 patients have highlighted significant metabolic alterations related to an increase in the glycolytic pathway to the detriment of the pentose phosphate pathway, highlighted by a characteristic increase in glucose consumption accompanied by an accumulation of intermediates of the glycolysis and higher levels of phosphofructokinase (PFK), the rate-limiting enzyme of glycolysis [24]. The metabolic shift towards ATP production in the glycolytic pathway clearly, also influences other metabolic pathways, such as the pentose phosphate pathway (PPP). In these conditions, the non-oxidative phase of PPP is increased, in which glycolysis intermediates are converted into the ribose-5-phosphate required for the synthesis of the nucleic acids, which is necessary for the virus replication [25,26]. In addition, high levels of oxidized glutathione and a significant decrease in enzymes forming part of the cellular defense line against oxidative stress were found, including superoxide dismutase 1 (SOD1), glucose-6-phosphate dehydrogenase, and peroxiredoxin [27,28,29]. RBCs from COVID-19 could, therefore, be more exposed to the attack of reactive oxygen species (ROS), resulting in induced cellular lysis and inability to carry oxygen. Recalling the important role played by the cytoplasmic domain of Band-3 as a regulator of erythrocyte metabolism in response to the different states of Hb oxygenation, it could be thought that the SARS-Band-3 bond alters the binding capacity of the cytoplasmic domain of Band-3 to glycolytic enzymes and deoxygenated Hb, causing the recorded metabolic irregularities [30,31]. Last but not least, it has been shown that Band-3 protein is associated with RBC membrane in a macro-complex that coordinates the shape of the cell (by the cytoskeletal proteins bound), carbon dioxide uptake (by the action of Carbonic anhydrase II bound to the C-terminal domain of Band-3), and the oxygen release from Hb (by the Bhor effect and by the direct binding between the cytoplasmic domain of Band-3 and deoxygenated Hb) [32]. In this context the attack of S1 Spike protein to Band-3 protein on the erythrocyte membrane [20] may affect the release of oxygen to metabolically active tissues.

## 3. Hemoglobin and SARS-CoV-2

Coronavirus, similar to other viruses, is able to interact with protoporphyrin IX through the spike protein. The interaction takes place between the beta chains of Hb, ORF 8, and the surface glycoproteins of the virus [33,34]. Liu et al. highlighted a number of viral proteins (orf1ab, ORF3a, ORF7a, ORF8a, and ORF10) as potential ligands to the 1-beta chains of hemoglobin [33]. This binding causes the Hb denaturation and the inhibition of viral replication by blocking (as also seen for other viruses) the SARS-CoV-2-cell fusion mediated by the spike protein [33,35]. Hb alteration caused by the viral proteins, decreasing the percentage of fully functional Hb in oxygen transport, could contribute to the development of hypoxia and multi-faceted syndrome, one of the main signs of COVID-19. The involvement of Hb beta chains in the evolution of COVID-19 finds an objective confirmation in some studies on the potential use of umbilical fetal blood transfusion on COVID-19 patients [36]. Increasing HbF in critically ill patients could help control disease progression, minimize morbidity, and increase survival rates [37]. Furthermore, the effect of SARS-CoV-2 on Hb has led to an assessment of COVID-19 as a potentially acquired acute porphyria [38]. Juan et al. reported an abnormal accumulation in the serum porphyrin profile of COVID-19 patients of the by-products uroporphyrin I, coproporphyrin I, and the metabolite coproporphyrin III, determined by high-performance liquid chromatography [39]. The interaction between SARS-CoV-2 and Hb would take place on two fronts: at the erythrocyte level, where the virus is introduced at the intracellular level through the link between spike and Band-3 protein, and at the level of the bone marrow, where the virus interacts with nascent erythroblasts through CD147 and CD26 [40,41]. The difference is that, at the erythrocyte level, the virus enters the red blood cell and interacts with the Hb molecule, but its replication is prevented by the absence of a nucleus while, in erythroblasts, the presence of nuclear material would facilitate viral replication and, in this case, the normal recycling of red blood cells from the spleen to the bloodstream would be inhibited, causing anemia [42]. It is important to note that high levels of glycosylated Hb increase CD147 expression, with an increased risk of further complications [43]. Lower Hb levels have been reported in several studies conducted in patients with severe COVID-19 disease, although there is no experimental evidence to date to support an alteration of the oxygen dissociation curve [44,44,45,46,47,48,49,50,51]. The decrease in Hb level might be a predictor of worsening pneumonia in COVID-19 patients, associated with the need for treatment with mechanical ventilation. 

As the disease worsens, other hematological markers associated with hemoglobin become altered, such as bilirubin and ferritin, which progressively increase [52,53]. Several researchers show elevated methemoglobin (MetHb) and carboxyhemoglobin (COHb) concentrations in severely ill patients’ blood and suggest these former as potential markers of disease severity [54,55,56]. In addition to the obvious effect of oxidizing drugs, the increase in MetHb formation may derive from a physiological reaction due to the increase in nitric oxide (NO) caused by acute anemia as part of the physiological reaction to the disease [44,57,58,59,60]. In particular, in hospitalized COVID-19 cases, there has been an increase in cases of anemia equal to about 60–70%. The release of NO is important for vasodilation and to prevent tissue hypoxia, but, at the same time, the NO release causes the oxidation of Hb in MetHb [55,56,57,61,62]. The increased level of CO-Hb could instead be related to the normal accumulation in the serum of porphyrin recorded in COVID-19 patients and to the progressive increase in bilirubin potentially linked to hemolytic anemia [38,39,52,53,56]. To this picture is added the breathing difficulty typical of COVID-19 that leads to a deficient CO elimination and potential formation of COHb.

## 4. Anemia and Iron Dysmetabolism in COVID-19

Anemia could be linked to iron homeostasis dysmetabolism found in subjects who suffered from severe or critical COVID-19 [63]. In detail, Sonnweber et al. highlighted persistent hyperferritinemia and alterations of iron homeostasis in non-resolving lung pathologies and poor patients’ performance [64]. Lanser et al. found the decline in Hb levels was more pronounced when accompanied by hyperferritinemia in hospitalized patients; transferrin levels decreased within the first week in all patients [63]. Many researches highlight low levels of Hb and high levels of ferritin in non-surviving patients [52,65,66]. 

Viruses generally increase iron deposits to promote their spread to host cells, and the immune system tends to control the overload of iron through transferrin saturation [67]. In COVID-19 patients, the virus invasion causes immune activation and the release of inflammatory cytokines such as interleukin 6, interleukin 1 beta, and tumor necrosis factor alpha [64]. Cytokines directly affect iron metabolism, triggering the production of hepcidin, the main hormone responsible for iron homeostasis, whose synthesis is increased by inflammatory cytokines and in cases of iron overload [68]. The release of hepcidin should instead be physiologically decreased in cases of anemia and hypoxia [69]. Ehsani also found a similarity (a conservative motif rich in cysteine) between the cytosolic subunit of the viral spike protein and hepcidin; SARS-CoV-2 would seem to mimic the hepcidin action [70]. Hepcidin binding to ferroportin limits the iron availability, blocking its export from cells; during COVID-19, there is, in fact, an iron overload in cells and tissues and a concomitantly reduced level of serum iron [65,69]. The reduced availability of serum iron results in a low transferrin saturation ratio that affects the Hb synthesis and erythropoiesis (RBCs production), leading to anemia of inflammation [69,71]. This decreased circulation of RBCs perpetuates hypoxemia and prevents tissue oxygenation, which is already difficult in patients with acute COVID-19 respiratory syndrome [72]. In fact, alterations in iron metabolism are associated with hypoxemia in COVID-19 patients [45,73]. 

## 5. Hemolysis and Oxidative Stress in COVID-19

Additional harmful processes that can occur in COVID-19 include a general increase in oxidative stress. During viral infection, neutrophils rapidly move towards the target tissues, where they release ROS in an oxidative burst necessary for eradicating phagocytosed viruses [74]. The increase in ROS favors a local inflammatory response that, if not well controlled, can cause oxidative stress phenomena that, in turn, perpetuate the activation of neutrophils, triggering a very dangerous chain reaction. Violi et al. showed that NADH oxidase2 (NOX2) is overexpressed in hospitalized COVID-19 patients [75]. The activation of NOX is one of the major factors contributing to the formation of ROS and the consequent increase in oxidative stress, inflammatory response, and increased severity of COVID-19 [76]. Lungs are the organs mainly involved in COVID-19, where the virus also causes edema, inflammation, and a decrease in oxygenation exacerbated by oxidative stress. Oxidative stress also plays a significant role in damaging the RBCs that can be impaired by becoming dysfunctional. High ROS level causes damage mainly to the erythrocyte membrane where polyunsaturated fatty acids are oxidized, leading to profound changes in the distribution of membrane lipids. These alterations affect the oxygen and carbon dioxide exchange and the deformation capacity of RBCs favoring thrombotic events observed in critically ill patients with COVID-19 [77]. In addition, the free heme, resulting from hemolysis present in the bloodstream of COVID-19 patients associated with hyperferritinemia, contributes to endothelial damage and the remodeling of pulmonary vessels [78]. The attack of SARS-CoV-2 proteins facilitates the removal of iron from the heme prosthetic group, leading to the loss of functional hemoglobin and release of free iron ions that, on the one hand, contribute to the increase in ferritin and, on the other, inexorably feed the reactions of Fenton and Haber–Weiss and oxidative stress [49,79]. Such oxidative imbalance causes mitochondrial dysfunction, further perpetuating the inflammatory state and hyperstimulating the release of cytokines; the process may culminate with ferroptosis associated with multiorgan oxidative stress in later stages of the disease [52,80]. Numerous data confirm the development of oxidative stress in viral infections [81,82]. There is evidence of a link between decreased expression of the antioxidant enzyme superoxide dismutase 3 (SOD3) in the lungs of elderly patients with COVID-19 and disease severity [83]. In addition, a low level of glutathione (GSH) and downregulation of glutathione peroxidase (GPX4) gene expression in SARS-CoV-2 infection was found [52,84,85,86,87]. In detail, in the RBCs from COVID-19 patients, Thomas et al. identified a high level of the end product of the PPP that suggests a high degree of oxidative stress also evidenced by an increase in oxidized glutathione (GSSG) [24]. To this oxidative picture is added a decrease in the most significant enzymes in the cells antioxidant response, a reduced level of peroxiredoxins (PRDX), superoxide dismutase 1 (SOD1), and glucose-6-phosphate dehydrogenase (G6PD) is registered. All these results unequivocally underline in RBCs from COVID-19 patients an increase in ROS level that may be linked to the increased aggregation and abnormal morpho-physiology observed by Renaux et al. [88]. In fact, ROS react with membrane lipids and proteins, producing lipid peroxidation and modified membrane proteins, resulting in phosphatidylserine exposure on the RBC surface. This membrane rearrangement involves an imbalance in cation homeostasis and a concomitant decrease in deformability [89]. When the endothelium is damaged by oxidative stress, RBCs may adhere to the injured matrix, contributing to thrombin generation predisposing the patient to thrombosis [90]. These alterations can contribute to thromboembolic complications and coagulopathies characteristic of some critically ill COVID-19 patients.

## 6. Intra and Extra-Erythrocytic NO Levels in COVID-19 Patients

Nitric oxide (NO) is a free radical gas that transmits a regulatory signal produced by the endothelium whose main biological activity is to induce relaxation of the aortic vascular walls. It also plays a role as a neurotransmitter and a defense in inflammatory processes. It is produced by an enzymatic reaction catalyzed by nitric oxide synthase (NOS), present in the body under three different isoforms: neuronal (nNOS), inducible (iNOS), and endothelial (eNOS) [91]. 

eNOS is mostly expressed in endothelial cells with an important role in blood pressure regulation. The NO’s small size and lack of charge facilitate its entry through the RBC membrane, where it is rapidly inactivated by oxyhemoglobin via conversion to MetHb and nitrate [92]. Under low oxygen saturation intra-erythrocyte, NO can also react with deoxyhemoglobin generating nitrosyl Hb. NO levels are significantly reduced in COVID-19 patients: this is likely related to vascular dysfunction and inflammation [87,88]. Although SARS-CoV-2, mainly infects the cells of the bronchial epithelium and pulmonary, residual viral particles have also been found in endothelial cells, leading to apoptosis and endothelial decrease in NO [78,93]. However, in the RBCs of COVID-19 patients, the increased levels of NO could be related to the silent hypoxia highlighted in some cases of severe disease [60,94]. In this context, the high levels of intraerythrocytic NO would contribute to inhibiting the amount of oxygen released at the tissue level [95,96]. Reduced serum NO levels could be caused by hemolysis of RBCs, following viral infection and subsequent Hb release. In this case, Hb and free heme would rapidly bind to NO, generating MetHb and nitrates and contributing to hypertension and vasoconstriction, elements both found in patients with severe COVID-19. NO inhibits blood clotting and excessive platelet activation, so a decrease in endothelial NO production could be related to potential thrombotic events and coagulation disorders in COVID-19 patients, characterized by a significant increase in D-dimer and risk of disseminated intravascular coagulation [97,98]. The hypothesis of a role of NO in the etiology of COVID-19 is accredited by the high risk of incurring severe COVID-19 disease for those patients suffering from diabetes, hypertension, and other cardiovascular diseases [99]. These pathologies are, in fact, often associated with decreased NO levels or decreased NO availability [100].

## 7. COVID-19 and Long-Term Changes

Several studies show that 50-70% of patients after 2 to 6 months still have a variety of symptoms due to SARS-CoV-2 infection [101,102]. This condition, known as “Long COVID”, is characterized by a general state of malaise; recovered patients often experienced headaches, concentration disorders, weakness, muscle fatigue, and shortness of breath [103,104]. In this context, Pasini et al. showed that, two months after the acute phase of the COVID-19 infection, some patients continued to have an altered hematological status [104]. In detail, there are very high serum levels of ferritin and D-dimer, accompanied by significantly low levels of Hb and albumin and a high degree of erythrocyte sedimentation. Kubancova et al. found significant phenotypic changes in RBCs from COVID-19 recovered patients. RBCs are less deformable, smaller, and more heterogeneous in size and deformation [105]. These changes in physical properties could be related to the alterations in the structural proteins and RBC membrane highlighted by Thomas et al. [24]. The persistence of RBCs, less deformable in recovered patients, could explain the symptomatology detected in Long COVID, especially considering that the RBC’s life average is estimated at 120 days [106]. This hypothesis is supported by the results of Kubancova et al. that identify phenotypic changes in blood cells even several months after SARS-CoV-2 infection [105].

## 8. Erythrocyte Morpho-Functional Changes in COVID-19

Considering the exponential progression of the SARS-CoV-2 pandemic, it is of great importance to take accurate and timely prognostic information to optimize patient management. In this context, the hematological investigation with a wide spectrum of analyses can be a valid routine support for COVID-19 patient monitoring, who, as demonstrated, share a series of alterations that may be related to the progression and severity of the disease. COVID-19 causes significant changes in the size and rigidity of RBCs; a decrease in the hematocrit level and increased RBCs amplitude referred to as RDW (red blood cell distribution width) has been recorded [107]. RDW is an indicator of the heterogeneity degree of erythrocyte size distribution in the blood, conventionally known as anisocytosis and used as an index of anemia [108,109]. A high RDW is considered a negative value for the diagnosis of different pathologies, such as pulmonary disease, liver and kidney failure, and cardiovascular disease [110,111,112,113]. In COVID-19, with the aggravation of the disease, the hematocrit and the concentration of Hb tend to progressively decrease while RDW increases progressively in the opposite direction. It is estimated that the RDW increase raises the chances of incurring the COVID-19 aggravation by about 9 times, and they are 16 times more likely to have severe acute kidney injury [114]. Therefore, RDW can be interpreted as an indicator of both disease severity and kidney damage in patients with SARS-CoV-2. RDW increase is an index of profound deregulation of RBC homeostasis that can result in impaired erythropoiesis and abnormal RBC survival, both caused by several metabolic alterations, including oxidative stress, inflammation, hypertension, dyslipidemia, hemolysis, and impaired erythropoietin function. Many of these metabolic alterations have been found in severe COVID-19. In particular, RBCs abnormalities recorded in COVID-19 patients may have multiple causes, including: alterations in iron homeostasis that could cause impaired erythropoiesis and accelerated release of circulating RBCs; fragmentation of proteins such as β-spectrin, ankyrin, Band-3 (at the N-terminal level), and lipid metabolism abnormalities, which could contribute to the morphological abnormalities; oxidative stress, which could contribute to altered flexibility and morphology identified in RBCs [24,44,69,71,73,115].

## 9. Nutrients and Natural Bioactive Molecules in the Treatment of COVID-19

Based on the mechanisms of action of the virus against the RBCs, the search for new molecules to counteract the evolution of the infection and contain the damage caused could be directed towards the molecules of natural origin, such as vitamins with antioxidant effect (vitamin C, E, A) or vitamin D which supports the immune system, enhancing the cell response against the inflammatory state caused by viral infection [116,117]. In this context, a balanced diet providing an adequate intake of carbohydrates, lipids, proteins, vitamins, and minerals (such as selenium zinc, iron, copper, and magnesium) can play a key role in increasing immune defenses. While on the contrary, dietary imbalances, such as a diet low in essential nutrients or too rich in fat, associated with a high risk of obesity, can increase the risk of complications of viral infections. In fact, a higher body mass index was found to be associated with an increased risk of hospitalization and severity of the disease [118,119]. In particular, the nanoencapsulation of some polyphenols, including caffeine, quercetin and cocoa flavanols, could provide new ideas to deal with the pandemic [120]. In fact, some flavanols are particularly efficient in counteracting ROS due to the presence of two OH groups on the B ring in catechol-type arrangement, supported also by the presence of a hydroxyl group in the 3-position and a double bond between atom carbon C-2 and C-3 of ring C that play a fundamental role in ROS scavenging [121]. Resveratrol, present in red wine and grapes, has been shown to have antioxidant properties and protective activity from Hb oxidation [122,123]. Caffeine, present in coffee and tea, has been shown to inhibit RBC membrane derangement by blocking caspase-3 activation and avoiding the production of carbonate and nitrogen dioxide, particularly harmful to the RBC membrane [124]. 

## 10. Conclusions

COVID-19 causes several alterations in hematological parameters. These blood abnormalities, if strictly monitored, could become a valid “sentinel” for the control of the evolution of the disease. It has been demonstrated that such blood changes are significantly more common and prominent in patients with severe COVID-19, and they are related to the progression and severity of infection. Thus, blood parameters could become an important potential biomarker for hospitalized patients and could be a valuable contribution to the management of the pandemic. In addition, the strong involvement of oxidative stress in the evolution of viral inflammation in RBCs suggests a possible involvement of nutraceutical and adequate diets as a potential strategy to avoid the cascade of events following SARS-CoV-2 infection, due to their strong involvement in counteracting the damage caused by oxidative stress. 
